# Curvilinear Magnonic Crystal Based on 3D Hierarchical Nanotemplates

**DOI:** 10.1021/acs.nanolett.5c06216

**Published:** 2026-01-23

**Authors:** Gianluca Gubbiotti, Olha Bezsmertna, Oleksandr V. Pylypovskyi, Rui Xu, Stéphane Chiroli, Fatih Zighem, Claudia Fernández González, Andrea Sorrentino, David Raftrey, Daniel Wolf, Axel Lubk, Peter Fischer, Damien Faurie, Denys Makarov

**Affiliations:** † CNR-Istituto Officina dei Materiali (IOM), 06123 Perugia, Italy; ‡ 28414 Helmholtz-Zentrum Dresden-Rossendorf e.V., Institute of Ion Beam Physics and Materials Research, 01328 Dresden, Germany; § Kyiv Academic University, 03142 Kyiv, Ukraine; ∥ LSPM—CNRS, UPR 3407, Université Sorbonne Paris Nord, 93430 Villetaneuse, France; ⊥ Laboratoire Albert Fert, UMR 137, CNRS-Thales, 91767 Palaiseau, France; # Alba Light Source, MISTRAL beamline, Cerdanyola del Vallés, Barcelona 08290, Spain; ∇ Department of Physics, University of California, Santa Cruz, 95064, California, United States; ○ Materials Sciences Division, 1666 Lawrence Berkeley National Laboratory, Berkeley, 94720, California, United States; ◧ Leibniz Institute for Solid State and Materials Research, 01069 Dresden, Germany

**Keywords:** Magnonic crystals, magnonic band structure, curvilinear magnetism, 3D magnetic nanostructures, 3D hierarchical templates

## Abstract

Curvilinear magnetic nanostructures enable control of magnetization dynamics through geometry-induced anisotropy and chiral interactions, as well as magnetic field modulation. In this work, we report a curvilinear magnonic crystal based on large-area square arrays of truncated nanospikes fabricated by conformal coating of 3D hierarchical templates with permalloy thin films. Brillouin light scattering spectroscopy reveals an anisotropic band structure with multiple dispersive and folded Bloch-type dispersive spin-wave modes as well as nondispersive modes exhibiting direction-dependent frequency shifts and intensity asymmetries along lattice principal axes. Finite element micromagnetic simulations indicate that curvature-induced variations of the demagnetizing field govern the magnonic response, enabling the identification of modes propagating in nanochannels and others localized on nanospike apexes or along the ridges connecting adjacent nanospikes. The combination of geometric curvature and optical probing asymmetry produces directional dependence of magnonic bands, establishing 3D hierarchical templates as a versatile platform for curvature-engineered magnonics.

## Graphical abstract

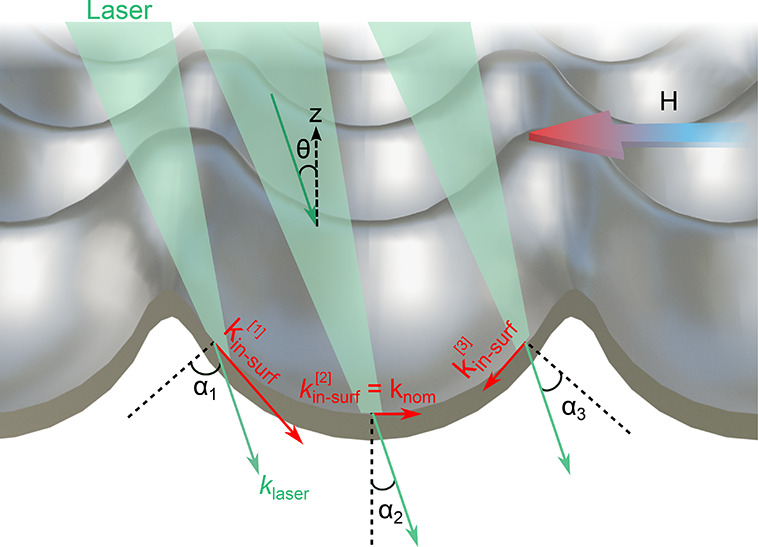

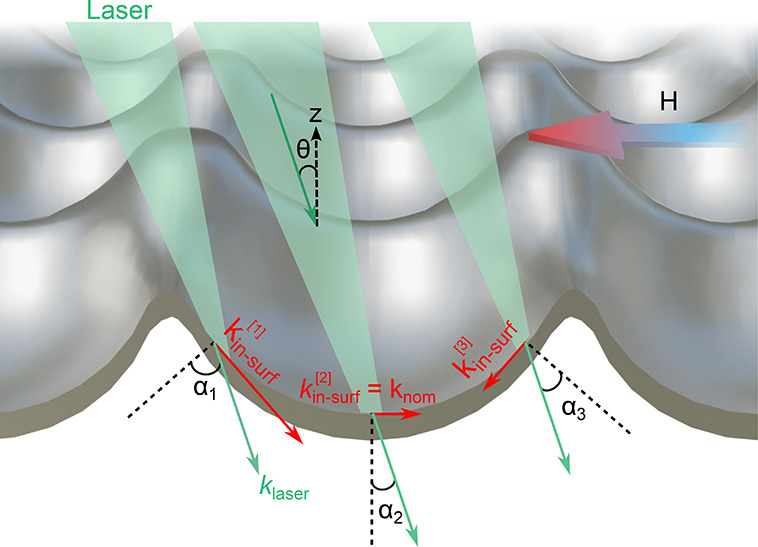

3D magnonic crystals—magnetic metamaterials with periodically modulated properties on the nanoscale—have gained increasing attention due to their potential to revolutionize information processing and wave-based computing.^1^ Unlike traditional 1D and 2D magnonic structures,^2–4^ 3D architectures offer enhanced control over spin-wave (SW) propagation, richer band structures, novel nonreciprocal and interference phenomena, and engineered SW pathways, which are key phenomena to developing energy efficient all-magnon circuits for neuromorphic computing and reconfigurable, multifunctional magnonic devices.^5–10^ Recent activities on 3D magnetic nanostructures were extended to studies of magnonic effects in complex-shaped samples using Brillouin light scattering (BLS) spectroscopy and ferromagnetic resonance (FMR) techniques. There are numerous theoretical and experimental explorations of SW phenomena in complex architectures like wireframes,^9,11,12^ nanowires,^13–16^ magnetic thin shells,^17^ periodic segments,^5,6,18^ waveguides with the broken translational symmetry,^19^ and meander-shaped magnonic crystals.^20–23^ So far, 3D magnonics has focused on the design of plane and straight segments as well as networks of interconnected magnonic waveguides and, therefore, ignored the effects of non-negligible geometric curvature.^24^ Curvilinear magnonics explicitly benefits from the effects of geometric curvature like anisotropic and chiral responses in the spirit of curvilinear and 3D magnetism.^18,25,26^ Despite being still in an early stage, the topic of curvilinear magnonics has already developed appealing theoretical predictions yet has not been accessed experimentally. It has been shown that finite tangential magnetostatic charges lead to nonreciprocity in SW propagation along a tube.^27–29^ A 1D curvilinear magnonic crystal designed by a periodically alternating curvature develops band gap edges determined by the effective geometric potentials.^30^ Recent studies reveal the role of the sample topology and a curvature-induced Berry phase in SW dynamics.^31,32^ A development of magnetic hierarchical nanostructures^33^ offers a possibility to extend the experimental framework of the curvilinear magnetism^18^ on curvilinear magnonics and unlock new functionalities for next-generation spintronic and magnonic devices.

Here, we report the first curvilinear magnonic crystal based on 3D hierarchical nanotemplates consisting of truncated nanospike structures arranged in a square lattice covered by soft magnetic pemalloy thin films. We measured the magnonic band structures (i.e., frequency vs wave vector) by using wave vector-resolved BLS spectroscopy for two orientations of the externally applied magnetic field along the high symmetry directions, i.e., [10] and [11] directions of the square lattice. The experimental results are supported by numerical simulations, which accurately reproduce the anisotropic magnonic band structure and enable the assignment of the spatial profiles and localization characteristics of the experimentally measured SW modes. We identify several intrinsic features of curvilinear magnonic crystals, including symmetry-broken excitation of SWs and asymmetric intensity between Stokes and anti-Stokes sides of the spectrum. The excitation range and intensity of the spectrum branches are determined by the curvature profile of the geometry. The curvilinear magnonic crystal supports a large number of localized modes originating from the sharp bends of the geometry.

We fabricated a curvilinear magnonic crystal using an anodized aluminum oxide template method.^34^ The aluminum foil is anodized and etched after nanoimprinting that defines a hierarchical square pattern without defects over several cm^2^ with nanoscale features of 50 nm-wide plateaus and a periodicity of *a* = 400 nm. The oxidized substrate is covered by a Permalloy (Ni_81_Fe_19_=Py) layer via magnetron sputtering (Figure 1a). Scanning electron microscopy (SEM) images of the final geometry of the sample are shown in Figure 1b–d. The obtained curvilinear 30 and 50 nm-thick ferromagnetic membranes form regular square lattices of truncated spikes with a height of 150 nm separated by hemispherical valleys. The distance between spikes along the [10] ([11]) direction is *a* = 400 nm ( $\sqrt{2}a=566$ nm). Using a focused ion beam (FIB) cut inclined under an angle of 5° to the [11] axis of the lattice (Figure 1b,d) we reconstruct the geometric structural unit of the lattice (Figure 1f–h). The spikes of a lateral size of 90 nm are connected by smooth 30 nm-wide ridges.The distance between spikes following the curved surface along the [10] ([11]) direction is $2l_{\mathrm{arc}}^{\left[10\right]}=454$ nm ( $2l_{\mathrm{arc}}^{\left[11\right]}=746$ nm) (Figure 1g,h). In the following, we refer to the in-plane and out-of-plane directions as the directions within the *xy*-plane and along the $\overset{⃗}{ẑ}$ -axis, respectively (Figure 1f–h). The in-surface directions are locally tangential to the curvilinear membrane and the out-of-surface direction coincides with the membrane normal $\overset{⃗}{\mathrm{n̂}}$ (Figure 1h). The integral magnetic properties of the curvilinear Py membranes are characterized by vibrating sample magnetomery (VSM). The in-plane hysteresis loops show a coercivity of about 2.5 mT (Figure 1e). The two branches of the hysteresis merge above a field of 10 mT, indicating the elimination of the domain pattern. The gradual increase in magnetic moment is attributed to a continuous competition of the Zeeman energy and the inhomogeneous shape anisotropy that prevents a strictly uniform magnetization distribution in any field. In particular, the in-surface shape anisotropy^35,36^ results in the magnetic component along the $\overset{⃗}{ẑ}$ axis (Figure 1f) at the inclined walls of the template. This magnetic state with a gradually varying magnetic moment within the structural unit of the lattice is a specific feature of curvilinear magnonics. Magnetic transmission soft X-ray microscopy (MTXM), which is sensitive to the magnetization component parallel to the photon propagation direction (thus, to the inclined walls of the membrane), indicates that the remanent state consists of periodic zigzag stripe domains with 90° domain walls between them that follow the geometric features of the lattice (Figure 1i). Off-axis electron holography at a valley shows that the in-plane magnetization ${\mathrm{M⃗}}_{xy}$ keeps a high local uniformity after application of an out-of-plane magnetic field corroborating the dominating impact of shape anisotropy at zero field (Figure 1j,k, Supporting Figure 3). To avoid complications with the analysis of BLS spectra due to the presence of magnetic merons,^33^ in the following, we focus on the magnetization dynamics in 30 nm-thick Py membranes.

**Figure 1:**
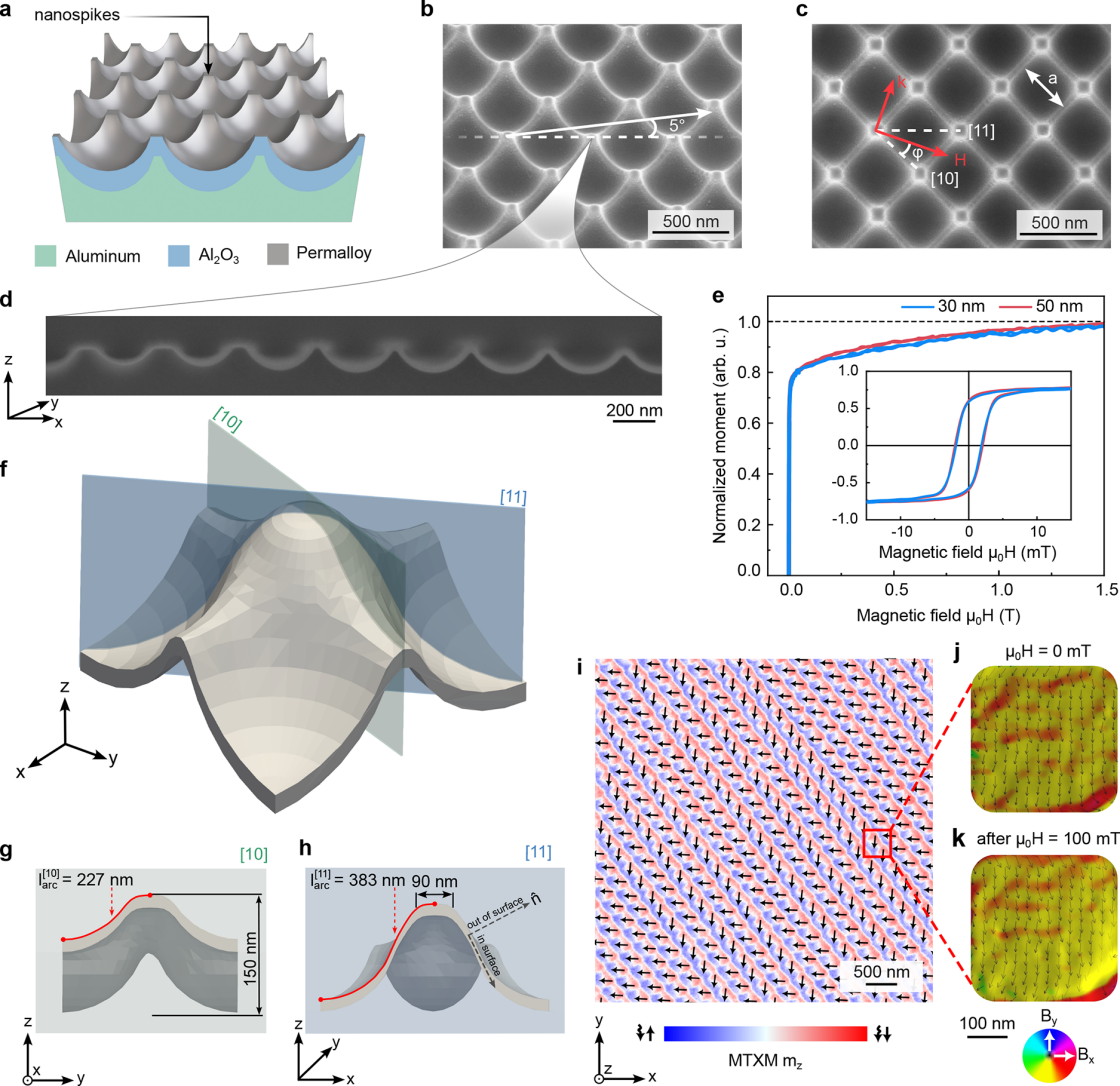
(a) Schematics of a curvilinear hierarchical template. Zoomed-in SEM images of (b) tilted and (c) top views of a 30 nm-thick Py film. Schematics in (b) indicates the position of the cross-section shown in (d) that was cut a small (5°) tilt angle of milling with respect to the diagonal of the square lattice. (e) *M*(*H*) hysteresis loops in an in-plane magnetic field for samples of 30 and 50 nm-thickness. The main panel shows the dependence at high field, low-field hysteresis are shown in the inset. Based on cross-sectional cut (d), the geometry of a single truncated nanospike is reconstructed (f). Parallel (g) and diagonal (h) cross sections of the reconstructed geometry, showing the lateral dimensions of a single nanospike. (i) Map of the out-of-plane magnetization component measured with MTXM of a 50 nm-thick sample, revealing formation of zigzag-like domains at remanence. (j,k) Mapping of the in-plane components of the projected magnetic induction (*B*_*x*_, *B*_*y*_) within a single nanoindentation between spikes reconstructed by off-axis electron holography: (j) initial remanent state, (k) remanent state after the sample was exposed to a 100 mT out-of-plane magnetic field.

Due to the absence of conservation of the normal component of the SW vector $k_{n}\overset{⃗}{\mathrm{n̂}}$ in metallic (opaque) materials,^37^ a laser beam interacting with a curvilinear magnonic crystal probes SWs with a range of in-surface wave vectors

1 $$k_{\mathrm{in}‐\mathrm{surf}}\left(\mathrm{r⃗}\right)=\frac{4\pi }{\lambda_{\mathrm{laser}}}\sin\left[\theta \pm \theta_{0}\left(\mathrm{r⃗}\right)\right]$$

where λ_laser_ is the laser wavelength (532 nm), θ is the incidence angle with respect to $\overset{⃗}{ẑ}$ , and $\theta_{0}\left(\mathrm{r⃗}\right)=\arccos\left(\overset{⃗}{\mathrm{n̂}}\cdot \overset{⃗}{ẑ}\right)$ is the inclination of the normal $\overset{⃗}{\mathrm{n̂}}$ at the coordinate r⃗ (Supporting Figure 4). However, the wave vector measured in our BLS experiments, *k*_nom_, corresponds to the in-plane projection of *k*_in‑surf_, which follows the curvilinear geometry, onto the *xy* plane. As a result, the projection of the Brillouin zone (BZ) observed in BLS does not match the intrinsic BZ experienced by the spin waves traveling along the curvilinear magnonic crystal and is determined by the in-plane periodicity of the lattice.

The spectra of thermally excited SWs are measured at room temperature using BLS spectroscopy in a backscattering configuration.^38^ A magnetic field H⃗ is applied in the sample plane perpendicular to the incidence direction of the light that defines the direction of the SW wave vector involved in the scattering process. Consequently, the measurements are conducted in the magnetostatic surface wave configuration, also called the Damon–Eshbach (DE) configuration,^39^ where the projection ${\mathrm{k⃗}}_{xy}$ is oriented perpendicular to the in-plane applied magnetic field H⃗ . The sample is mounted on a goniometer, enabling rotation around the field direction to vary the incidence angle of light θ from 0° to 70°. We note that the curvilinear geometry leads to the appearance of shadows for θ ≳ 30° in the detection solid angle for scattering light, thus limiting asymmetrically the upper boundary of the range of excited wave vectors according to eq ([Disp-formula eq1]). In the following, we focus on the analysis of the SW propagation for the external magnetic field applied along the [10] and [11] directions of the square lattice. The experimental data are discussed together with the simulation results. The numerical analysis is based on a finite element method (FEM) that solves the Landau–Lifshitz–Gilbert (LLG) equation in the frequency domain providing eigenfrequencies and spatial mode profiles.

Figure 2 presents analysis of the SW propagation for a magnetic field μ_0_ *H* = 50 mT (above coercive field, see Figure 1e) applied along the [10] direction. The spectra shown in Figure 2a display well-resolved peaks observed on the Stokes (S) and anti-Stokes (AS) sides corresponding to SWs with the opposite *k*-vectors (i.e., positive *k* for AS and negative *k* for S sides of the BLS spectra). To follow the evolution of the peak frequencies with wave vectors characterizing different SW modes, the peaks have been highlighted by different colors. These modes are displayed with the same color in Figure 2b and overlaid with the simulated dynamic magnetization, which have been carried out using micromagnetic modeling (see Supporting Information). The corresponding calculated mode profiles are shown in Figure 2d-g. The overall band structure is characterized by the presence of dispersive modes with frequencies that are periodic in reciprocal space (Brillouin zones), induced by the lattice periodicity, a typical behavior of Bloch-type waves propagating in magnonic crystals.

**Figure 2:**
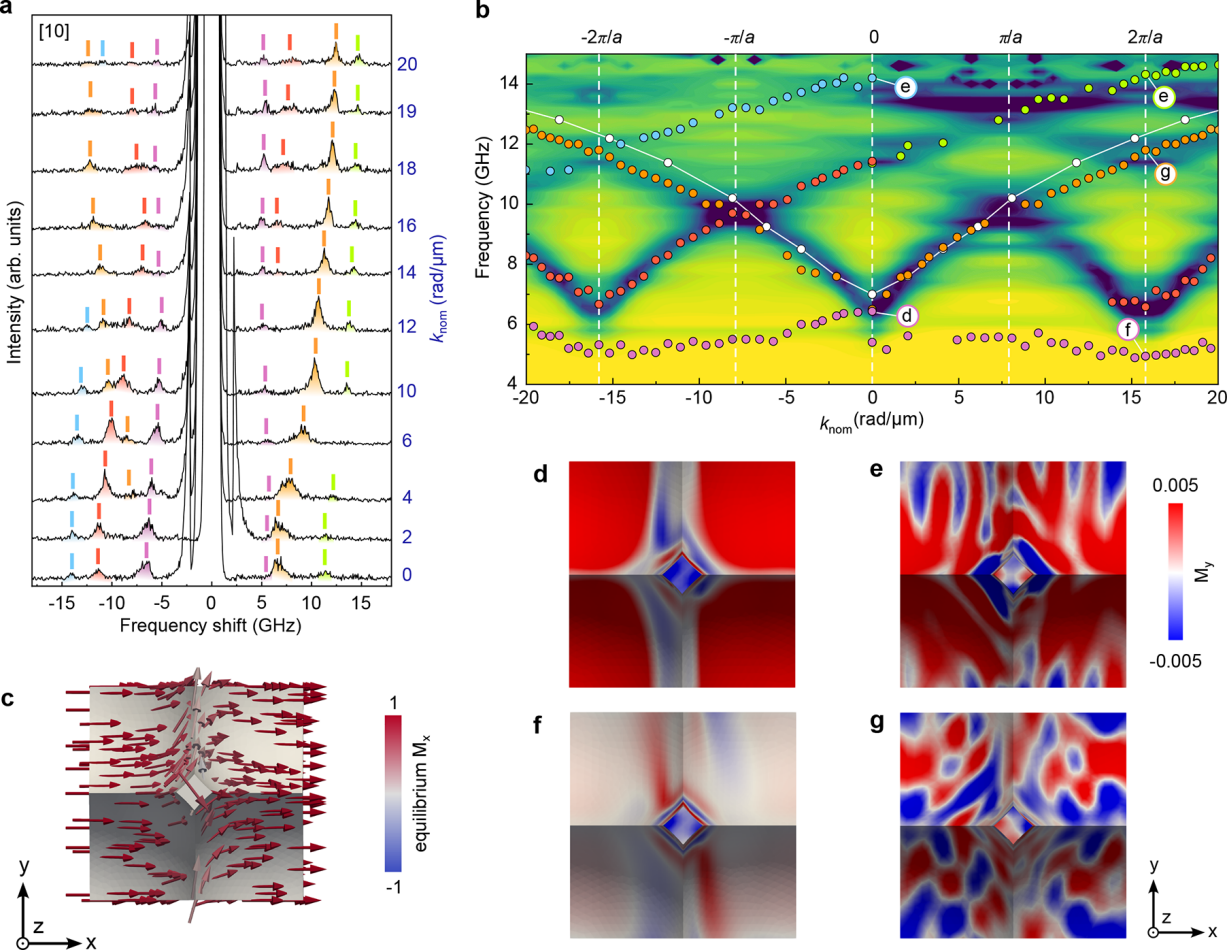
(a) Sequence of measured BLS spectra for different in-plane wave vector *k*_nom_ with magnetic field of 50 mT applied along the [10] direction. Spectra are acquired in the Damon–Eshbach geometry with a 50 mT in-plane magnetic field. Labels near each spectrum correspond to the *k*_nom_-value expressed in rad/μm. (b) Experimental SW dispersion measured by BLS (colored symbols) in the same experimental condition of (a). The white symbols correspond to the dispersion of a planar 30 nm-thick reference film. The colored background represents simulated dynamic magnetization amplitudes. Vertical dashed lines indicate positions of the Brillouin zones. (c) An equilibrium magnetization distribution over a structural element of the lattice shows a tilt of the magnetization on the inclined facets due to competition between external field and shape anisotropy. The state is simulated in a field of 50 mT. (d–g) Spatial distribution of the dynamic magnetization component *M*_*y*_ for the modes labeled (d–g) in panel (b), illustrating the evolution from quasi-uniform to strongly localized oscillations. Low-frequency modes (d,e) are confined near the apex of truncated nanospikes, while higher-frequency modes (g,f) extend over the flanks and valleys with alternating phase between neighboring cells, indicating the formation of a magnonic band through the Bragg reflection.

As *k*_nom_ increases, the mode frequencies and the intensities of the peaks evolve progressively yet in a distinct manner on the S and AS sides (Figures 2a and 2b). On the S side, the lowest-frequency resonance (purple peak at around 6 GHz) shifts monotonically toward lower frequencies as *k*_nom_ increases up to approximately 12 rad/μm. For higher *k*_nom_ values, this mode also appears on the AS side and becomes nearly dispersionless, i.e., independent of *k*_nom_, with a frequency of about 5 GHz. Additional peaks (red and light blue) are observed at higher frequencies on the S side of the spectra. These peaks do not have a counterpart on the AS side, and their frequencies decrease with increasing *k*_nom_. On the AS side, the most intense peak (orange) can be continuously tracked over the entire *k*_nom_ range with its frequency increasing as *k*_nom_ increases.

The mode colored in orange (Figures 2a and 2b) corresponds to a quasi-uniform precession of the magnetization similar to the DE mode of a planar reference film (white symbols in Figure 2b). Although slightly altered by the topography, the quasi-uniform mode extends over the full unit cell of the lattice and remains the most intense and continuous feature in the spectra, appearing predominantly on the S side due to the surface nonreciprocity inherent to the DE geometry. Its profile is presented in Figure 2g.

The mode colored in violet (Figures 2a,b) is localized at the apex and upper ridges of nanospikes, where the influence of the demagnetizing field is maximum, leading to a weakly dispersive and relatively confined excitation; see the magnetization profile in Figure 2c. The simulated mode profile (Figure 2f) confirms that the precession amplitude is concentrated near the apex of the truncated spike, with nodes appearing progressively along the flanks as the frequency increases.

The other modes shown in Figure 2a,b are altered by the curvilinear profile of the magnetic 3D template. The branch colored red is reintroduced through band folding and forms a higher-order standing-wave pattern across adjacent nanospikes. It shows a systematic offset between the S and AS sides pronounced in the mode intensity. This asymmetry originates from the curvature-induced variation of the optical probing geometry driven by the distribution of *k*_in‑surf_ for the given excitation angle. As a result, the S and AS signals probe slightly different optical momenta, leading to apparent duplication or lateral shifts of certain branches. The modes colored green and blue in Figure 2a,b have similar behavior. Their relative intensity and frequency offset vary systematically with the scattering geometry. Because these modes are confined to inclined regions, they are particularly affected by the variation of the local optical wave vector, indicating that curvature couples asymmetries of magnetic and optical responses.

The analysis of BLS spectra and their comparison with simulations for the case of the SW propagation along the [11] direction is given in Figure 3. We observe multiple peaks of nonequal intensity confirming that several thermally populated SW modes coexist even close to the center of the BZ. The S side exhibits a rich and sharp peak structure with numerous narrow peaks spanning the 6...13 GHz range. While the AS side also displays multiple resonances, those are broader and less intense compared to the peaks on the S side of the spectra. The frequency evolution of the purple, red, and blue peaks is similar to what was observed for the [10] direction. Remarkably, their frequencies shift downward by approximately 2 GHz compared to their values in the [10] orientation. This suggests a strong dependence of the SW dynamics on the magnetic field direction, likely due to modifications in the internal magnetization configuration and the effective field landscape within the sample as well as the curvature-induced coupling with the incoming laser beam. In contrast, there is an additional low-intensity peak shown in green that remains nearly constant at around 5 GHz.

**Figure 3:**
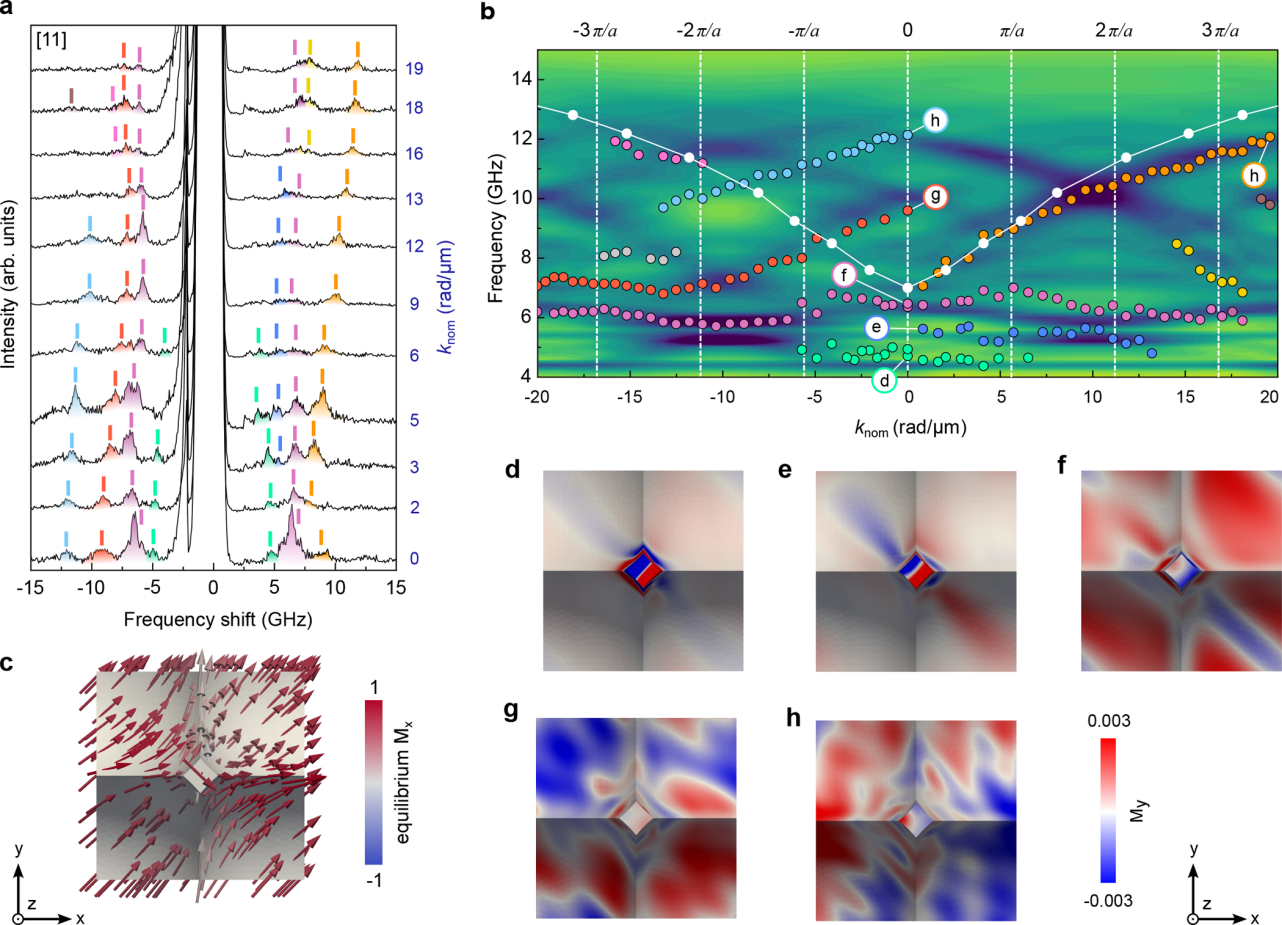
(a) Sequence of measured BLS spectra for different in-plane wave vector *k*_nom_ with magnetic field of 50 mT applied along the [11] direction. Spectra are acquired in the Damon–Eshbach geometry with a 50 mT in-plane magnetic field. Labels near each spectrum correspond to the *k*_nom_-value expressed in rad/μm. (b) Experimental SW dispersion measured by BLS (colored symbols) along the [11] propagation direction. White symbols correspond to the dispersion of a planar 30 nm-thick reference film. The colored background shows results of micromagnetic simulations, representing the calculated amplitude of the dynamic magnetization *M*_*y*_ as a function of frequency and in-plane wave vector *k*_nom_. Vertical dashed lines indicate positions of the Brillouin zones. (c) Simulated equilibrium magnetization configuration *M*_*x*_ within one unit cell of the lattice highlighting the gradual reorientation of the magnetization along the inclined facets. (d–h) Spatial maps of the dynamic magnetization component *M*_*y*_ for selected modes labeled (d–g) in panel (b). Low-frequency modes (d,e) are mainly localized at the apex and top edges of the nanospike. Higher-frequency modes (f–h) extend over the lateral flanks and valley regions, forming alternating phase patterns between neighboring cells. This is characteristic of the magnonic band formation and mode hybridization.

As follows from Figure 3b, the agreement between simulations and the experiment is more direct. At *k* = 0, several branches coexist in the 5...10 GHz range, revealing that the lateral surface modulation lifts the degeneracy of the fundamental excitation even at the center of the BZ. The most intense and dispersive mode indicated in orange (this mode is analogous to the DE mode of the planar film) increases with *k*_nom_ (Figure 3b,h). As a result of the band folding, this mode continues on the S side of the spectra (shown in red in Figure 3b,g).

The mode colored in blue (Figure 3b,h), which is barely visible experimentally, exhibits an alternating phase between neighboring cells, consistent with a weak Bragg folding. Comparing with the [10] case, here the projection of the laser beam on opposite facets is more symmetric leading to a better correspondence between the mode intensities on the S and AS sides of the spectra. The observed spectral complexity for the SWs propagating along the [11] direction is thus dominated by the intrinsic magnonic hybridization rather than extrinsic optical geometry effects.

We analyzed the impact of an applied in-plane magnetic field on the mode frequencies (Supporting Figure 8). The magnetic field was applied along the [10] and [11] directions of the square lattice (Supporting Figure 8a,b). These measurements were performed at a fixed wave vector |*k*_nom_| = 4.1 rad/μm, corresponding to an angle of incidence of 10°. For a magnetic field applied along the [10] direction and for positive field values, all of the observed peaks in the spectra appear exclusively on one side, regardless of the wave vector *k*_nom_. When the direction of the applied field is reversed, the frequency shifts of all modes also reverse, changing from negative to positive values and *vice versa*. For the propagation along the [10] direction, the frequencies of all the observed peaks decrease monotonically starting from +50 mT, reaching a minimum at zero applied field, and subsequently increase as the field is further applied in the opposite direction up to –50 mT (Supporting Figure 8c). A similar trend is observed when the magnetic field is applied along the [11] direction (Supporting Figure 8d). In this case, some peaks appear on only one side of the spectrum without a corresponding peak on the opposite side. For example, the peak marked by the orange segment is observed only on the AS side of the spectra for the positive field and shifts to the S side when the field direction is reversed to negative values.

In summary, we fabricated a curvilinear magnonic crystal of square symmetry based on hierarchical templates with truncated nanospikes connected by narrow ridges. The spin wave dynamics along the [10] and [11] directions was analyzed by means of Brillouin light scattering and finite element micromagnetic simulations. A peculiar feature of the geometry is the competition between the external magnetic field and in-surface anisotropy that leads to periodic modulation of the magnetization distribution even in strong magnetic fields. We identified a pronounced curvature-induced magneto-optical coupling. In addition to common features of 3D magnonic crystals, we found that a continuous variation of the curvature of the sample broadens the range of wave vectors and introduces geometry-driven asymmetry between the Stokes and anti-Stokes sides of the spectra. The apex of truncated nanospikes and ridges support dispersiveless modes due to the variation of magnetization and related stray fields.

Our results demonstrate that curvilinear hierarchical templates constitute a scalable platform to tailor magnonic dispersion and control nonreciprocal SW transport. In particular, the demonstrated possibility to realize freestanding curvilinear magnetic nanomembranes and their transfer onto a substrate of choice^33^ would enable postfabrication tunability of the properties of curvilinear magnonic crystals. For instance, transferring these membranes onto piezoelectric substrates^40,41^ could offer tunability of the crystal symmetry and periodicity by an electric field. Alternatively, transferring magnetic 3D nanomembranes onto polymer substrates^42,43^ could allow for controlled modifications by mechanical deformations. In this respect, this work opens new opportunities for designing curvature-engineered magnonic devices for prospective low-power- and wave-based information processing.

## Supplementary Material



## Notes

Detailed descriptions of the 3D hierarchical template fabrication and thin film deposition, along with experimental details of (i) magnetic transmission X-ray microscopy (MTXM), (ii) electron holography, (iii) Brillouin light scattering (BLS) measurements (including magnetic field dependence), and (iv) the description of micromagnetic simulations (PDF)

## References

[1] Gubbiotti, G. , Ed. Three-dimensional magnonics: layered, micro- and nanostructures; Jenny Stanford Publishing, 2019.

[2] Gubbiotti G., Tacchi S., Madami M., Carlotti G., Adeyeye A. O., Kostylev M. (2010). Brillouin light scattering studies of planar metallic
magnonic crystals. J. Phys. D: Appl. Phys..

[3] Tacchi S., Gubbiotti G., Madami M., Carlotti G. (2017). Brillouin light scattering
studies of 2D magnonic crystals. J. Phys.: Condens.
Matter.

[4] Krawczyk M., Grundler D. (2014). Review and prospects of magnonic crystals and devices
with reprogrammable band structure. J. Phys.:
Condens. Matter.

[5] Sahoo S., May A., van Den Berg A., Mondal A. K., Ladak S., Barman A. (2021). Observation
of Coherent Spin Waves in a Three-Dimensional Artificial Spin Ice
Structure. Nano Lett..

[6] Guo H., Deenen A. J. M., Xu M., Hamdi M., Grundler D. (2023). Realization
and Control of Bulk and Surface Modes in 3D Nanomagnonic Networks
by Additive Manufacturing of Ferromagnets. Adv.
Mater..

[7] Cheenikundil R., d’Aquino M., Hertel R. (2025). Defect-sensitive high-frequency modes
in a three-dimensional artificial magnetic crystal. npj Computational Materials.

[8] Kumar C., Mondal A. K., Pal S., Mathur S., Scott J. R., van den Berg A., Adekunle O. A., Ladak S., Barman A. (2025). Magnetic Charge
State Controlled Spin-Wave Dynamics in Nanoscale Three-Dimensional
Artificial Spin Ice. arXiv (Mesoscale and Nanoscale
Physics).

[9] Guo, H. ; Lenz, K. ; Gołbiewski, M. ; Narkowicz, R. ; Lindner, J. ; Krawczyk, M. ; Grundler, D. Coherent Spin Waves in Curved Ferromagnetic Nanocaps of a 3D-printed Magnonic Crystal. arXiv 2025, 10.48550/arXiv.2506.1610 (accessed 2026-01-07). PMC12862454 41406381

[10] Birch, M. T. ; Fujishiro, Y. ; Belopolski, I. ; Mogi, M. ; Chiew, Y.-L. ; Yu, X. ; Nagaosa, N. ; Kawamura, M. ; Tokura, Y. Nanosculpted 3D helices of a magnetic Weyl semimetal with switchable nonreciprocity. arXiv 2025, 10.48550/arXiv.2506.17023 (accessed 2026-01-07). 41565782

[11] Sahoo S., Mondal S., Williams G., May A., Ladak S., Barman A. (2018). Ultrafast magnetization dynamics in a nanoscale three-dimensional
cobalt tetrapod structure. Nanoscale.

[12] Cheenikundil R., Bauer J., Goharyan M., d’Aquino M., Hertel R. (2022). High-frequency modes in a magnetic buckyball nanoarchitecture. APL Materials.

[13] Gallardo R. A., Alvarado-Seguel P., Landeros P. (2022). High spin-wave asymmetry and emergence
of radial standing modes in thick ferromagnetic nanotubes. Phys. Rev. B.

[14] Landeros, P. ; Ot́alora, J. A. ; Streubel, R. ; Ḱakay, A. Curvilinear Micromagnetism; Springer International Publishing, 2022; pp 163–213.

[15] Korber L., Verba R., Otalora J. A., Kravchuk V., Lindner J., Fassbender J., Kakay A. (2022). Curvilinear spin-wave dynamics beyond
the thin-shell approximation: Magnetic nanotubes as a case study. Phys. Rev. B.

[16] Brevis F., Landeros P., Lindner J., Kakay A., Korber L. (2024). Curvature-induced
parity loss and hybridization of magnons: Exploring the connection
of flat and tubular magnetic shells. Phys. Rev.
B.

[17] Gaididei Y., Kravchuk V. P., Sheka D. D. (2014). Curvature Effects in Thin Magnetic
Shells. Phys. Rev. Lett..

[18] Dobrovolskiy, O. V. ; Pylypovskyi, O. V. ; Skoric, L. ; Ferńandez-Pacheco, A. ; Van Den Berg, A. ; Ladak, S. ; Huth, M. Curvilinear Micromagnetism: From Fundamentals to Applications; Springer International Publishing, 2022.

[19] Martyshkin A. A., Beginin E. N., Stognij A. I., Nikitov S. A., Sadovnikov A. V. (2019). Vertical
Spin-Wave Transport in Magnonic Waveguides With Broken Translation
Symmetry. IEEE Magnetics Letters.

[20] Beginin E. N., Sadovnikov A. V., Sharaevskaya A. Y., Stognij A. I., Nikitov S. A. (2018). Spin wave
steering in three-dimensional magnonic networks. Appl. Phys. Lett..

[21] Gubbiotti G., Sadovnikov A., Sheshukova S. E., Beginin E., Nikitov S., Talmelli G., Adelmann C., Ciubotaru F. (2022). Spin-wave
nonreciprocity and formation of lateral standing spin waves in CoFeB/Ta/NiFe
meander-shaped films. J. Appl. Phys..

[22] Sakharov V. K., Beginin E. N., Khivintsev Y. V., Sadovnikov A. V., Stognij A. I., Filimonov Y. A., Nikitov S. A. (2020). Spin waves in meander
shaped YIG film: Toward 3D magnonics. Appl.
Phys. Lett..

[23] Gubbiotti G., Sadovnikov A., Beginin E., Nikitov S., Wan D., Gupta A., Kundu S., Talmelli G., Carpenter R., Asselberghs I., Radu I. P., Adelmann C., Ciubotaru F. (2021). Magnonic Band
Structure in Vertical Meander-Shaped Co_40_Fe_40_B_20_ Thin Films. Physical Review
Applied.

[24] Gubbiotti G. (2025). 2025 roadmap on 3D nanomagnetism. J. Phys.:
Condens. Matter.

[25] Makarov D., Volkov O. M., Ḱakay A., Pylypovskyi O. V., Budinsḱa B., Dobrovolskiy O. V. (2022). New Dimension in Magnetism and Superconductivity:
3D and Curvilinear Nanoarchitectures. Adv. Mater..

[26] Xu M., Deenen A. J. M., Guo H., Morales-Ferńandez P., Wintz S., Zhakina E., Weigand M., Donnelly C., Grundler D. (2026). Geometry-induced spin chirality in a non-chiral ferromagnet
at zero field. Nat. Nanotechnol..

[27] Otalora J. A., Yan M., Schultheiss H., Hertel R., Kakay A. (2016). Curvature-Induced Asymmetric
Spin-Wave Dispersion. Phys. Rev. Lett..

[28] Sheka D. D., Pylypovskyi O. V., Landeros P., Gaididei Y., Ḱakay A., Makarov D. (2020). Nonlocal chiral symmetry breaking in curvilinear magnetic
shells. Communications Physics.

[29] Otalora J. A., Yan M., Schultheiss H., Hertel R., Kakay A. (2017). Asymmetric spin-wave
dispersion in ferromagnetic nanotubes induced by surface curvature. Phys. Rev. B.

[30] Korniienko A., Kravchuk V., Pylypovskyi O., Sheka D., van den
Brink J., Gaididei Y. (2019). Curvature induced magnonic crystal
in nanowires. SciPost Physics.

[31] d’Aquino M., Hertel R. (2025). Nonreciprocal Inertial Spin-Wave Dynamics in Twisted
Magnetic Nanostrips. Phys. Rev. Lett..

[32] Thonikkadavan, A. ; d’Aquino, M. ; Hertel, R. Rotating Spin Wave Modes in Nanoscale M̈obius Strips. 2025. Mesoscale and Nanoscale Physics. https://arxiv.org/abs/2508.15463 (accessed 01-07-2026).

[33] Bezsmertna O., Xu R., Pylypovskyi O., Raftrey D., Sorrentino A., Fernandez-Roldan J. A., Soldatov I., Wolf D., Lubk A., Scḧafer R., Fischer P., Makarov D. (2024). Magnetic Solitons in
Hierarchical 3D Magnetic Nanoarchitectures of Nanoflower Shape. Nano Lett..

[34] Xu R., Zeng Z., Lei Y. (2022). Well-defined nanostructuring with
designable anodic aluminum oxide template. Nat.
Commun..

[35] Carbou G. (2001). Thin Layers
in micromagnetism. Mathematical Models and Methods
in Applied Sciences.

[36] Kohn R. V., Slastikov V. V. (2005). Another Thin-Film Limit of Micromagnetics. Archive for Rational Mechanics and Analysis.

[37] Sandercock J. R., Wettling W. (1979). Light scattering from surface and bulk thermal magnons
in iron and nickel. J. Appl. Phys..

[38] Carlotti G., Gubbiotti G. (2002). Magnetic properties of layered nanostructures studied
by means of Brillouin light scattering and the surface magneto-optical
Kerr effect. J. Phys.: Condens. Matter.

[39] Damon R., Eshbach J. (1961). Magnetostatic modes of a ferromagnet slab. J. Phys. Chem. Solids.

[40] Challab N., Faurie D., Haboussi M., Adeyeye A. O., Zighem F. (2021). Differentiated
Strain-Control of Localized Magnetic Modes in Antidot Arrays. ACS Appl. Mater. Interfaces.

[41] Chiroli S., Faurie D., Haboussi M., Adeyeye A. O., Zighem F. (2023). Magnetization
dynamics of elastically strained nanostructures studied by coupled
micromagnetic-mechanical simulations. Phys.
Rev. B.

[42] Faurie D., Adeyeye A. O., Zighem F. (2021). Prospects toward flexible magnonic
systems. J. Appl. Phys..

[43] Zighem F., Faurie D. (2021). A review on nanostructured thin films on flexible substrates:
links between strains and magnetic properties. J. Phys.: Condens. Matter.

