# Supplementary material for: Curvilinear Magnonic Crystal Based on 3D Hierarchical Nanotemplates
Source: Nano Lett. 2026 Jan 23;26(4):1561–8. doi: 10.1021/acs.nanolett.5c06216 (PMC13105353; doi:10.1021/acs.nanolett.5c06216)
Supplement: Supplementary file 1 [file nl5c06216_si_001.pdf]

Supporting information for
Curvilinear magnonic crystal based on 3D hierarchical nanotemplates

Gianluca Gubbiotti,[1,*] Olha Bezsmertna,[2] Oleksandr Pylypovskyi,[2,3] Rui Xu,[2] Stéphane Chiroli,[4,5]
Fatih Zighem,[4] Claudia Fernández González,[6] Andrea Sorrentino,[6] David Raftrey,[7,8]
Daniel Wolf,[9] Axel Lubk,[9] Peter Fischer,[7,8] Damien Faurie,[4] and Denys Makarov[2,†]

[1] *CNR-Istituto Officina dei Materiali (IOM), 06123 Perugia, Italy*
[2] *Helmholtz-Zentrum Dresden-Rossendorf e.V., Institute of Ion Beam Physics and Materials Research, 01328 Dresden, Germany*
[3] *Kyiv Academic University, 03142 Kyiv, Ukraine*
[4] *LSPM—CNRS, UPR 3407, Université Sorbonne Paris Nord, 93430 Villetaneuse, France*
[5] *Laboratoire Albert Fert, UMR 137, CNRS-Thales, 91767 Palaiseau, France*
[6] *Alba Light Source, MISTRAL beamline, Cerdanyola del Vallès 08290, Spain*
[7] *Department of Physics, University of California, Santa Cruz, 95064, California, USA*
[8] *Materials Sciences Division, Lawrence Berkeley National Laboratory, Berkeley, 94720, California, USA*
[9] *Leibniz Institute for Solid State and Materials Research, 01069 Dresden, Germany*

(Dated: January 9, 2026)

CONTENTS

I. Fabrication of a 3D hierarchical template 2

II. MTXM measurements 3

III. Electron holography measurements 4

IV. BLS measurements 5

V. Micromagnetic simulations 7
  A. Simulated hysteresis and equilibrium magnetic states 7
  B. Magnonic band structures simulations 10

VI. Magnetic field dependence in BLS 11

References 14

* gubbiotti@iom.cnr.it
† d.makarov@hzdr.de

I. FABRICATION OF A 3D HIERARCHICAL TEMPLATE

Fabrication of a large-area array of truncated spike-shaped nanostructures followed the approach described in [1, 2]. The sample preparation process is illustrated schematically in Supporting Figure 1. Initially, a surface-polished aluminum foil of 0.2 mm thickness was nanoimprinted using a nickel stamp decorated with square-lattice nanopillar pattern, leading to the formation of an array of nanoindentations (Supporting Figure 1a). The initial pores were then evolved at the sites of the prepatterned nanoindentations through anodization in an electrolyte solution with 3.86 wt.% H_3PO_4 for 1 min at a voltage of 160 V (Supporting Figure 1b). To further sharpen the spike geometry, an etching step was carried out in a 4.4 wt.% H_3PO_4 solution at 60°C for 6 min. Once the desired surface morphology was achieved (Supporting Figure 1c), layers of Permalloy of 30 and 50 nm thickness were deposited via magnetron sputtering (Supporting Figure 1d) from alloyed $Fe_{19}Ni_{81}$ target. Additionally, 5 nm of Ta was deposited as seed and capping layers. The deposition was carried out at room temperature in Scia Multi 300 sputtering system (scia Systems GmbH, Germany) using Ar as sputtering gas (base pressure: 5.0×10^{-8} mBar, deposition pressure 1.4×10^{-3} mBar). In addition to the patterned samples, a planar 30-nm-thick NiFe film was deposited under identical conditions and used as a reference for spin-wave dispersion measurements.

Scanning electron microscopy (SEM) images were taken using NVision hybrid system (Carl Zeiss & SII Nanotechnology). Top-view, tilted and cross-sectional images were taken at 5 kV accelerating voltage. For cross-sectional imaging, a selected area of the sample surface was first protected with an ion-beam-deposited Carbon layer (about 900 nm thick) to prevent any deformation of studied nanostructures. The cross section was then prepared by focused ion beam milling using a 60 µm aperture and a working distance of 5.2 mm.

A precise three-dimensional reconstruction of the investigated geometry (Figure 1f in the main text) was performed in the open-source software Blender, using the dimensions extracted from the SEM cross-sectional images.

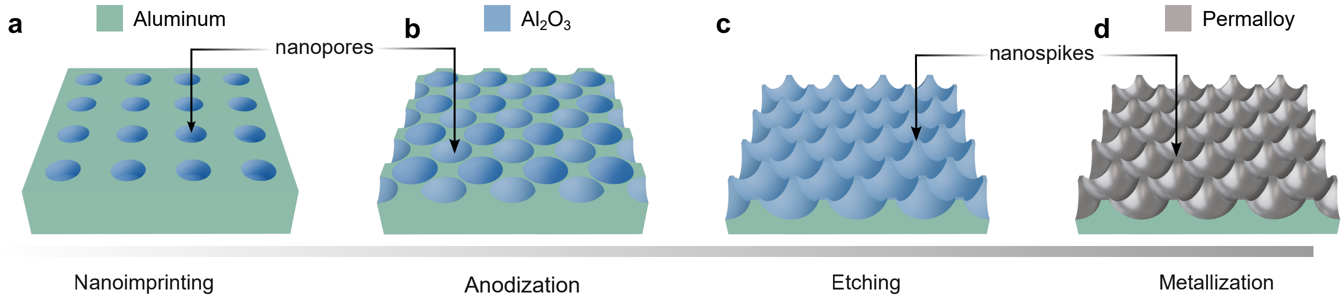

Supporting Fig. 1. Schematic of sample preparation. (a) nanoindentation with Nickel stamp; (b) anodization; (c) etching and (d) deposition of Permalloy film.

To avoid any complication with the interpretation of the BLS data due to the presence of noncollinear magnetic textures like vortices and antivortices [1], we deliberately focus on the magnetization dynamics studies in thinnest films (30-nm-thick).

II. MTXM MEASUREMENTS

Magnetic transmission X-ray microscopy (MTXM) experiments were performed at the full-field soft X-ray transmission microscope of the Mistral beamline at the ALBA Synchrotron [3]. Monochromatic, circularly polarized X-rays were generated by adjusting the electron orbit in the bending magnet source and selecting the desired wavelength with a variable line spacing plane grating monochromator, which provided a resolving power of approximately 2000 with a 15 μm exit slit. The beam was focused onto the sample using a single-bunch glass capillary. A Fresnel zone plate with an outermost zone width of 25 nm then acted as an objective lens, producing a magnified image (MAG = 1450; effective pixel size ≈ 9 nm) on a charge-coupled device (CCD) detector (Pixis XO 1024, Oxford Instruments), corresponding to a field of view of roughly 8.9 μm × 8.9 μm. All measurements were carried out under high-vacuum conditions. Images were acquired at the Fe L3 absorption edge to enhance magnetic contrast. After selecting the region of interest and fine-tuning the focus, thirty frames were recorded for each polarization state with an exposure time of 2 seconds per frame. Flat-field (FF) images, representing the incident beam intensity, were obtained by removing the sample from the beam; 60 frames were captured under the same conditions. Data preprocessing was performed using the "MARTApp" platform developed at Mistral [4]. The procedure for each polarization included: (i) converting the raw images into Nexus (HDF5) format, (ii) normalizing each frame with the corresponding flat-field data, and (iii) aligning and averaging the images. This resulted in two transmission images corresponding to the two circular polarizations (T_{C^+}, T_{C^-}). To compensate for any instability in the flat-field intensity following a polarization change, a correction factor was applied to one image so that the average intensity across the field of view matched that of the other. The aligned images were then combined to compute the X-ray magnetic circular dichroism (XMCD) signal using the standard formula:

$$\text{XMCD signal} = \frac{-\log T_{C^+} - (-\log T_{C^-})}{2} \tag{1}$$

In the resulting XMCD images, the observed contrast represents the projection of the local magnetic moment along the X-ray beam direction (Figure 1i in the Main text, Supporting Figure 2). Regions appearing in red correspond to magnetic moments aligned parallel to the beam, whereas blue areas indicate moments oriented antiparallel.

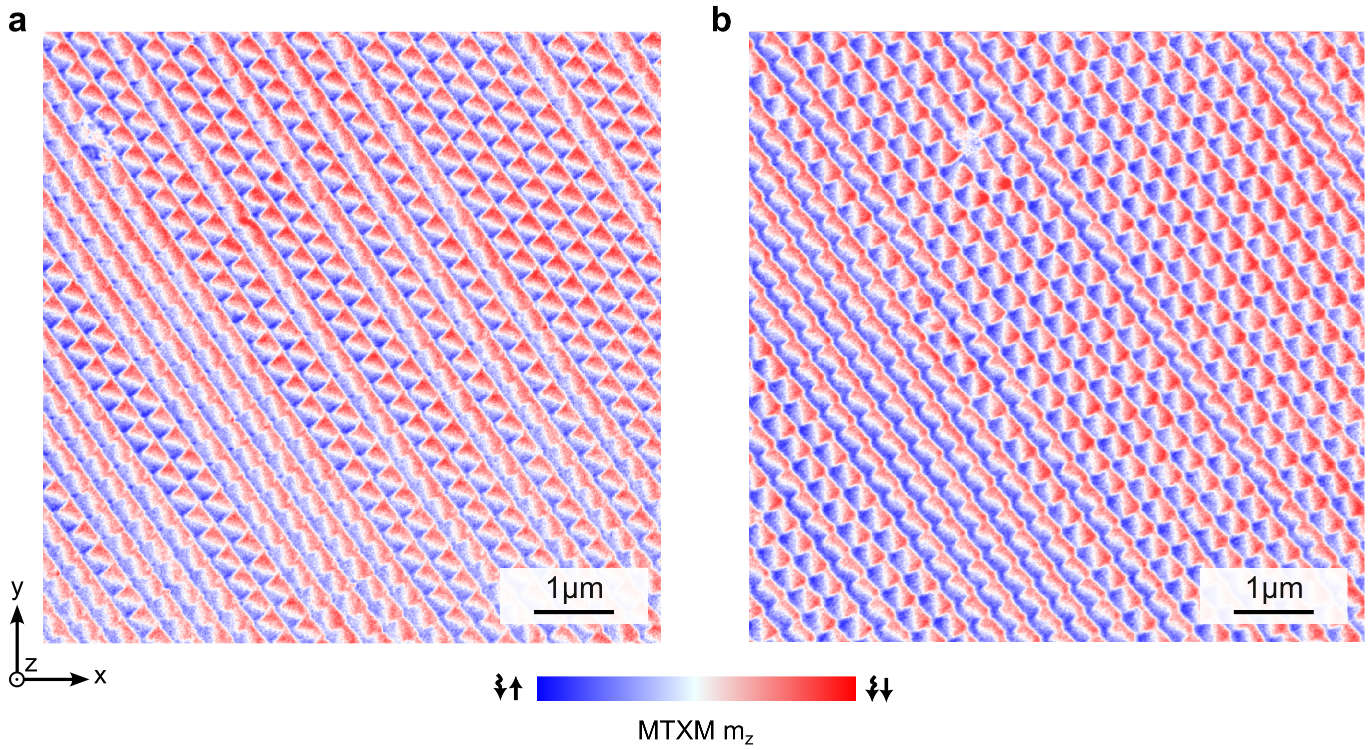

Supporting Fig. 2. XMCD contrast of different regions of the curvilinear freestanding nanostructure, depicting out-of plane magnetization component. Zig-zag domain structure is formed, similar to the one shown in main Figure 1i.

III. ELECTRON HOLOGRAPHY MEASUREMENTS

Off-axis electron holography was carried out following the procedure outlined by, e.g., Volkov et al.[5]. To this end, electron holograms of the valleys between truncated nanospikes were recorded using a double-corrected FEI Titan3 80-300 transmission electron microscope (ThermoFisher Comp., USA) operated at an acceleration voltage of 300 kV in magnetic field-free Lorentz mode (objective lens switched off). A fringe spacing of 1.4 nm with a 7 % fringe contrast was achieved in the electron holograms, which we acquired with a 4k×4k Gatan Oneview CMOS camera. Amplitude and phase images were reconstructed from the electron holograms. Subsequently, the sample was flipped up-side down outside the microscope and electron holograms of the identical position were recorded again. The phase images before and after sample flipping were further processed to separate electric and magnetic phase shifts, and calculate the final projected magnetic induction map (in-plane components). Supporting Figure S3 presents the electrostatic phase map which reveals the topology of one mesh of the truncated nanospikes.

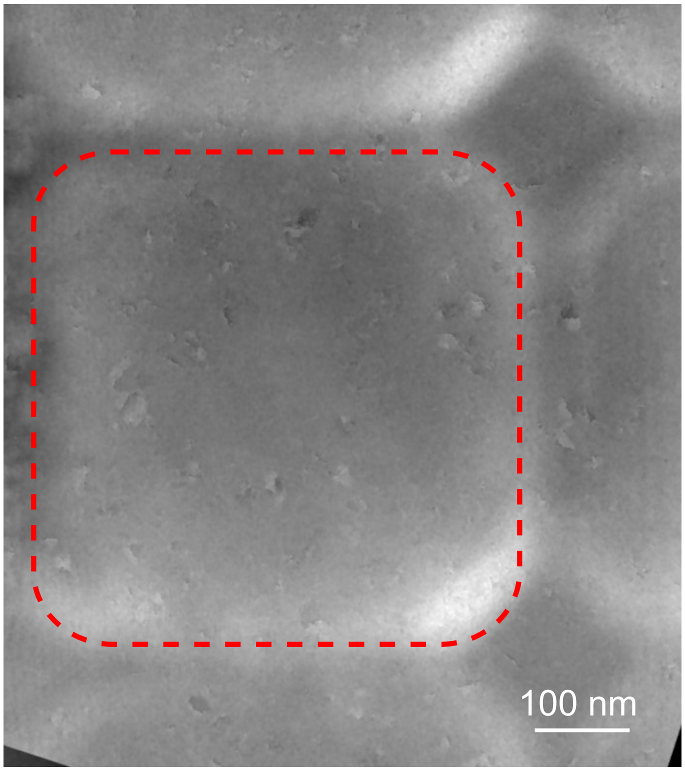

Supporting Fig. 3. (a) off-axis electron holography of one mesh of the truncated nanospikes. The electrostatic phase map illustrates the mesh's topology, where brighter regions indicate thicker portions and darker regions correspond to thinner portions. The red dashed box marks the area, where the magnetic induction maps depicted in Figures 1j,k of the main text were obtained.

IV. BLS MEASUREMENTS

The spectra of thermally excited spin-waves were measured at room temperature using the BLS spectroscopy in a backscattering configuration. A monochromatic laser beam from a solid-state laser ($\lambda_{\text{las}} = 532\,\text{nm}$) was focused onto the sample surface, by a camera objective with numerical aperture of NA = 0.24, and forming a spot with a diameter of approximately $30\,\mu$m. The backscattered light was analyzed in frequency using a tandem (3+3) Sandercock-type Fabry–Pérot interferometer [6]. The sample was mounted on a goniometer, enabling rotation around the field direction to vary the incidence angle of light θ with respect to $\hat{\boldsymbol{z}}$ from 0° to 70°. A magnetic field H was applied in the sample plane perpendicular to the incidence plane of the light which defines the direction of the spin-wave wave vector involved in the scattering process. Consequently, the measurements were conducted in the magnetostatic surface wave, also called the Damon-Eshbach (DE), configuration [7], where the spin-wave wave vector k_{nom} is oriented perpendicular to the in-plane applied magnetic field H.

We note that in curvilinear magnonic crystals, the band gap is determined by the real space periodicity of the path, which is traveled by the spin-wave [8]. Therefore, the important geometric parameter is the arclength between the neighboring truncated nanospikes of the template. For the [1 0] ([1 1]) direction, the arclength is $2l_{\text{arc}}^{[1\,0]} = 454\,\text{nm}$ ($2l_{\text{arc}}^{[1\,1]} = 746\,\text{nm}$) (Figure 1g,h in Main text), which are substantially different compared to the in-plane lattice periodicity of the template of $a = 400\,\text{nm}$ ($\sqrt{2}a = 566\,\text{nm}$). Hence, for a curvilinear magnonic crystal, the boundary of the Brillouin zone along the specific direction is defined along the curvilinear coordinate that follows the actual curvilinear sample geometry. As spin-waves propagate along a curved profile, their wave vector k_{nom} can be decomposed into in-plane $\boldsymbol{k}_{xy}$ and out-of-plane components $k\hat{\boldsymbol{z}}$. In metallic (opaque) materials, the out-of-surface component is not conserved, while the in-plane component is [9]. Therefore, due to the conservation of the in-surface momentum during the scattering process, the magnitude of the spin-wave wave vector can be described using the standard relation generally applied to planar structures [10], $k_{\text{in-surf}}(\boldsymbol{r}) = \frac{4\pi}{\lambda_{\text{las}}} \times \sin[\theta \pm \theta_0(\boldsymbol{r})]$, where $\theta_0(\boldsymbol{r})$ is the direction of the surface normal at the coordinate $\boldsymbol{r}$, see Supplementary Figure 4.

This aspect has major consequences on the interpretation of the BLS measurements compared to the theoretical analysis: (i) Because of continuous change in the surface normal $\theta_0(\boldsymbol{r})$, the single incidence angle θ excites a finite range of $k_{\text{in-surf}}$ across the geometric structural unit different from the nominal value $k_{\text{nom}} = 4\pi/\lambda_{\text{las}} \sin\theta$. (ii) The measured wave vector corresponds only to a geometric projection of the true quasi-momentum defined along the periodic path to xy plane. As a result, the Brillouin zone observed in BLS does not match the intrinsic Brillouin zone of the curvilinear magnonic crystal and is determined by the in-plane periodicity of the lattice, i.e. $a = 400\,\text{nm}$ ($\sqrt{2}a = 566\,\text{nm}$) for the [1 0] ([1 1]) direction.

To sum up, the real space periodicity is highly relevant for the discussion of the experimental results reported in our manuscript because of (i) the measurement technique projects the spin waves living on a curved manifold on xy plane and (ii) long waves near the center of the Brillouin zone. We anticipate that other methods like stripline excitations and detection in BLS, may access features directly related to the spin wave propagation along the curved geometries [8, 11].

We note that in the sequences of BLS spectra presented in Figure 2a and Figure 3a of the Main text we observe two types of peaks: (i) those that are symmetric in frequency on the Stokes and anti-Stokes side of the spectra (these are reciprocal spin waves) and (ii) those appearing only on one side of the spectra. Based on the data in hand, we believe that the apparent nonreciprocal features are mainly due to coupling mechanism of light with thermal magnons. Any curvature-induced nonreciprocity would require further characterization.

In this work, we focus primarily on the analysis of the peak frequencies rather than on their intensities. A systematic and quantitative investigation of the BLS cross-section is beyond the scope of the present paper and will require major additional calculation efforts. This is because the number and intensity of peaks observed in the BLS spectra depend not only on the sample curvature but also on the coupling mechanism between light and magnons, the spatial profile of modes and their interplay with the local curvature of the sample, as well as on the off-diagonal spin-spin correlation function, which contributes to the light scattering intensity in opaque materials [12].

The BLS measurements reported in the manuscript were taken at room temperature. Since we are detecting thermally excited spin waves without any external driving mechanism, lowering the temperature reduces the magnon population and, consequently, the BLS intensity. For this reason, room-temperature characterization performed at a fixed magnetic field allows for the observation of all possible magnon excitations and enables reconstruction of the magnonic band structure. The peaks detected by BLS correspond to modes with specific spatial profiles and wavelengths, compatible with the wavevector k selected by the BLS scattering geometry. This procedure was successfully applied both for planar [13, 14] and vertical magnonic crystals [15].

A shadowing effect of the laser beam by the geometry (Supporting Fig. 4) could potentially obscure some peaks at large k values. At the same time, the experimentally detected certain peaks at large k values indicate that the curvature-induced shadowing effect is not the only factor affecting the observation of the BLS peaks in curvilinear

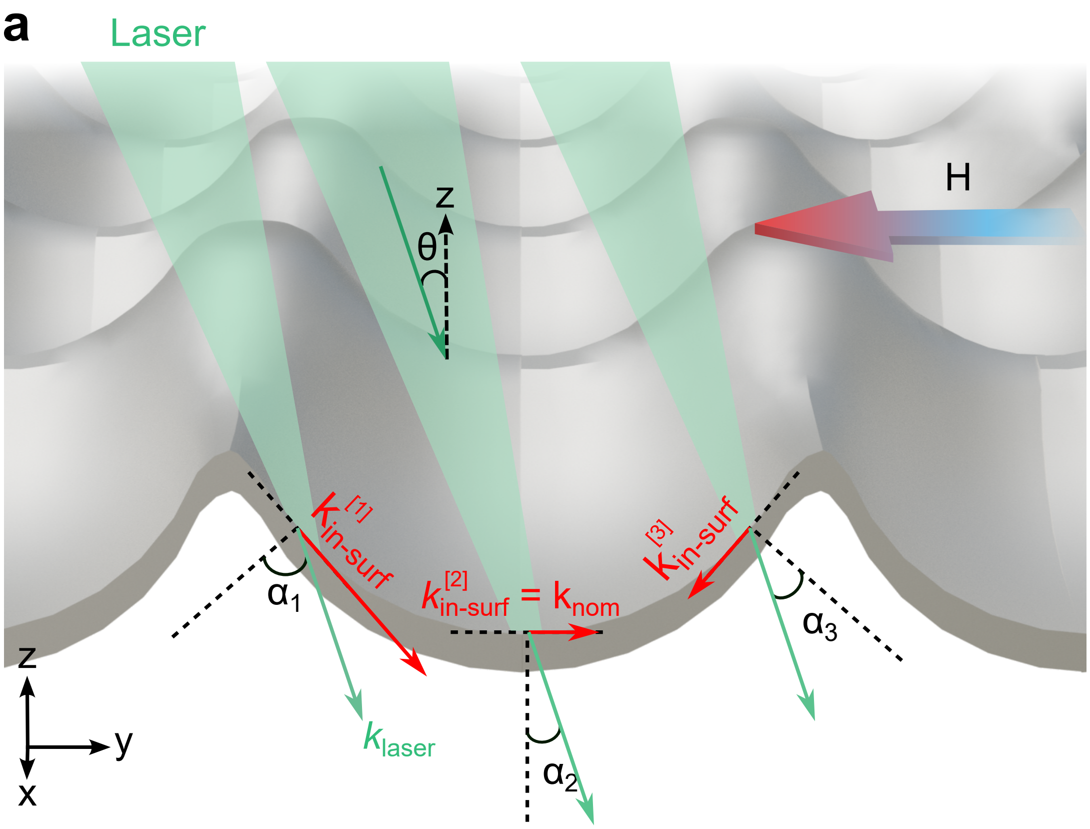

Supporting Fig. 4. Schematics of the detection of SWs in a curvilinear geometry. The laser beam oriented at the angle θ with respect to $\hat{\boldsymbol{z}}$, falls under the angle α to the local normal of the curved geometry. It detects SWs with $k_{\text{in-surf}}$ tangential to the surface. In the shown geometry, only $k^{[2]}_{\text{in-surf}} = k_{\text{nom}}$ which corresponds to a nominal excitation wave vector in BLS. The direction of the magnetic field $\boldsymbol{H}$ is indicated as well.

magnonic crystals. A systematic and quantitative investigation of the BLS cross-section is beyond the scope of the present paper where we focus on the anisotropic band structure observed for different orientations of the applied magnetic field.

V. MICROMAGNETIC SIMULATIONS

A. Simulated hysteresis and equilibrium magnetic states

To compute the quasi-static magnetic response of the truncated nanospike array, we performed finite-element micromagnetic simulations using COMSOL Multiphysics®. The finite-element (FEM) framework is particularly suitable for this system because it allows accurate discretization of the full three-dimensional geometry, including the truncated apex, inclined facets, and curved ridges connecting neighboring nanospikes, as reconstructed from experimental cross-sections. We simulate magnetic hysteresis loops for the geometry shown in Supporting Figure 5. The field is applied in-plane along [1 0] and [1 1] lattice directions (Supporting Figure 7a) and out of xy plane (Supporting Figure 7b).

The magnetization dynamics is governed by the Landau-Lifshitz-Gilbert (LLG) equation:

$$\frac{d\boldsymbol{M}}{dt} = -\gamma \boldsymbol{M} \times \boldsymbol{H}_{\text{eff}} + \frac{\alpha}{M_s} \boldsymbol{M} \times \frac{d\boldsymbol{M}}{dt} \tag{2}$$

where γ is the gyromagnetic ratio, α the Gilbert damping parameter, and M_s the saturation magnetization. The effective field is given by:

$$\boldsymbol{H}_{\text{eff}} = \boldsymbol{H}_{\text{app}} + \boldsymbol{H}_{\text{demag}} + \boldsymbol{H}_{\text{exch}}, \tag{3}$$

with

$$\boldsymbol{H}_{\text{demag}} = -\nabla\phi, \qquad \boldsymbol{H}_{\text{exch}} = \frac{2A}{\mu_0 M_s} \nabla^2 \boldsymbol{M}, \tag{4}$$

where ϕ is the magnetic scalar potential, A the exchange stiffness, and μ_0 the vacuum permeability.

Because COMSOL relies on a variational formulation, the LLG equation (2) is recast into its weak (finite-element) form by multiplying it with suitable vector test functions and integrating over the sample volume[16]. This weak formulation enables the incorporation of complex boundary conditions, curved geometries [17], and spatially varying magnetic fields [18], while ensuring numerical stability during relaxation toward equilibrium [19].

For the hysteresis simulations, the weak-form LLG is integrated in time under a slowly ramped external magnetic field until a stable equilibrium configuration is reached for each field value. The applied field is oriented along the principal symmetry directions of the square lattice, namely the [1 0] and [1 1] directions, in order to reproduce the experimental conditions.

To model the extended array, periodic boundary conditions were imposed along the two in-plane directions in the FEM mesh. At this stage, the periodicity is enforced through simple field continuity,

$$\boldsymbol{M}_{\text{dst}} = \boldsymbol{M}_{\text{src}}, \tag{5}$$

since static equilibrium states do not involve any propagation phase. Free boundary conditions were applied along the thickness. The resulting equilibrium magnetization states display the characteristic curvature-induced nonuniformities observed experimentally, including tilted magnetization along the nanospike sidewalls and modulated internal fields near the apex and ridges. These FEM-computed static configurations constitute the initial states for the dynamic band-structure calculations described in the next section.

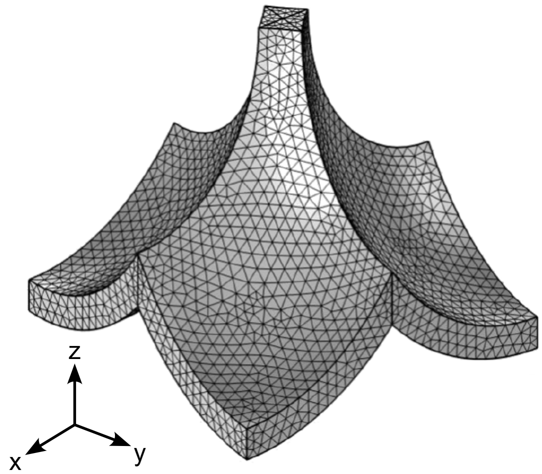

Supporting Fig. 5. Three-dimensional finite-element mesh used for the micromagnetic simulations, showing the full geometry of the curved structure in the (x, y, z) coordinate system.

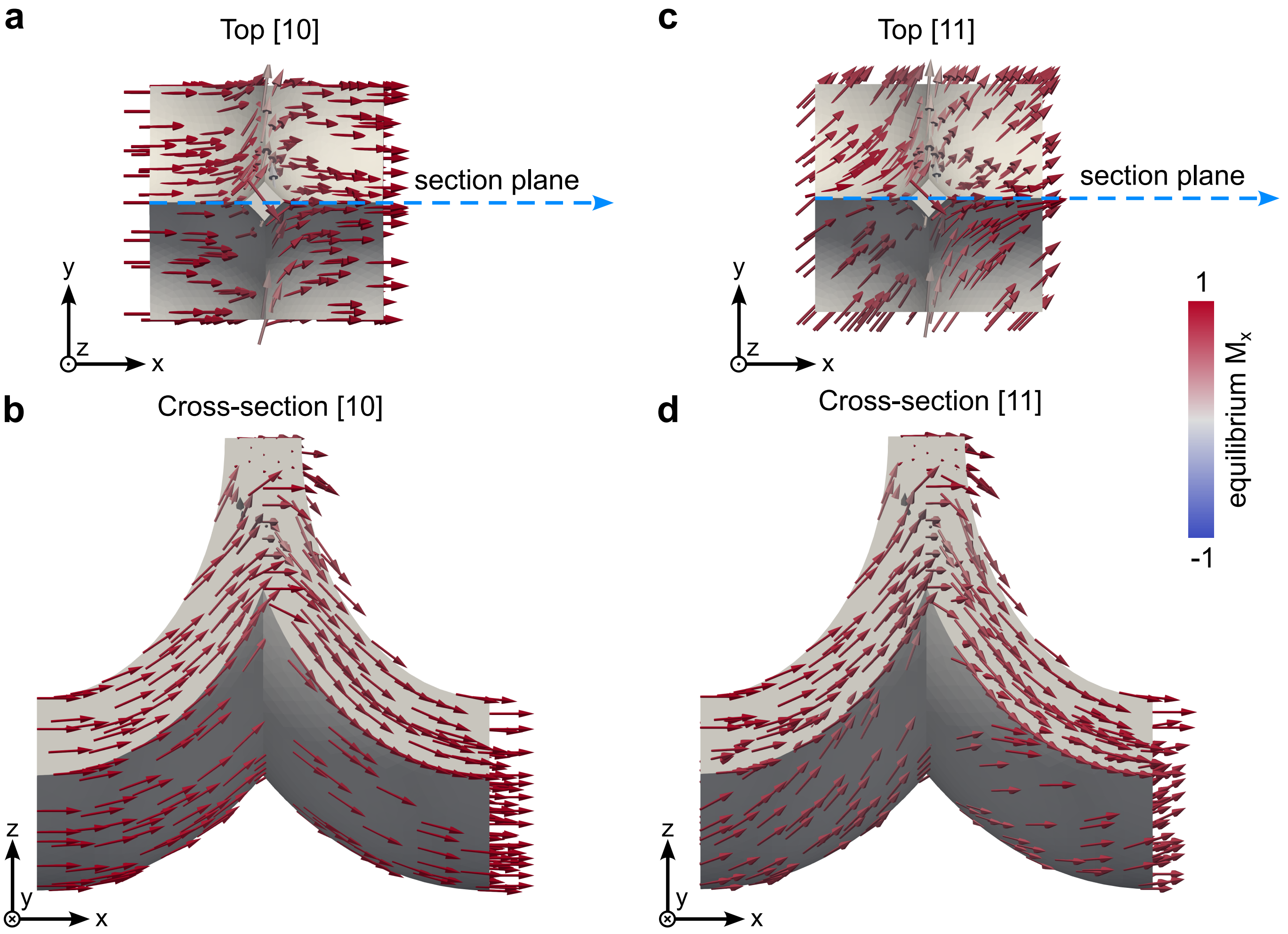

Supporting Fig. 6. Simulated equilibrium magnetization configurations within one unit cell. The simulation is done for the object exposed to magnetic field of 50 mT. Arrows denote the M_x component of magnetization. (a,c) Top view and (b,d) corresponding cross-section for the case when magnetic field is applied along (a,b) [1 0] and (c,d) [1 1] direction. The section planes are indicated by blue dashed lines in the top view panels (a,b).

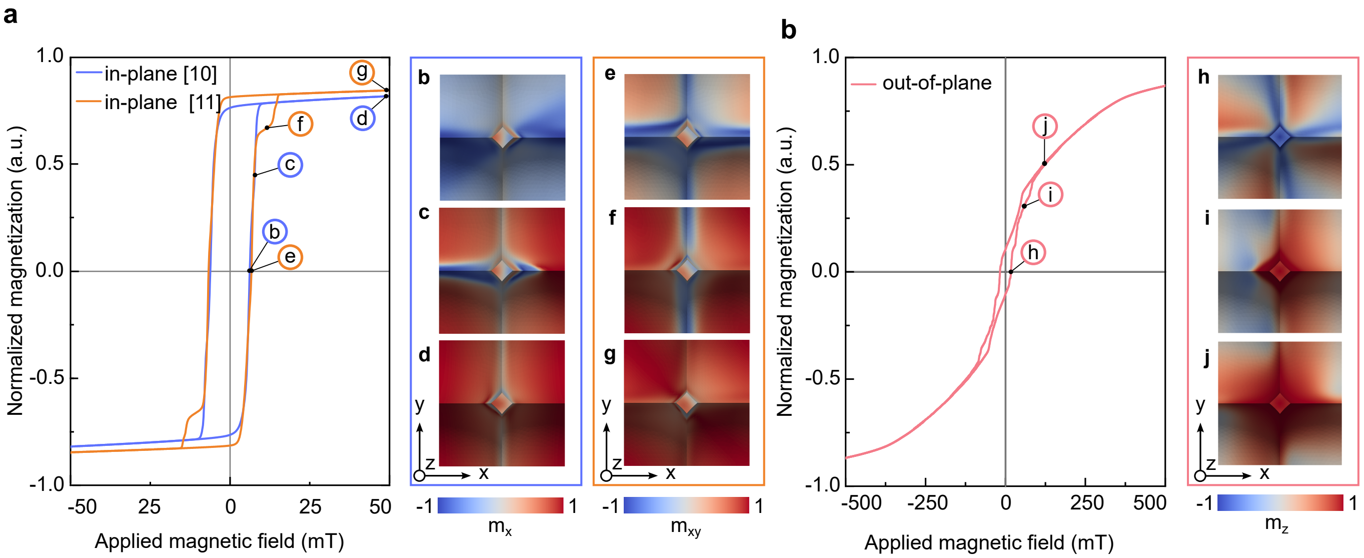

Supporting Fig. 7. (a) Simulated in-plane hysteresis loops for magnetic fields applied along the [1 0] and [1 1] directions. Two curves display the magnetization response as a function of the applied field for each direction. (d) Simulated hysteresis loop for a magnetic field applied along the out-of-plane $\hat{\boldsymbol{z}}$ direction, showing the corresponding evolution of magnetization with the applied field. Simulated magnetization states in (b-d), (e-g) and (h-j) represent several characteristic magnetization states of the pyramid.

B. Magnonic band structures simulations

The magnonic band structures of the truncated nanospike array were obtained by solving a finite-element eigenfrequency problem derived from the weak-form LLG equation [20]. Starting from the static equilibrium magnetization configurations computed in the previous section, we consider small dynamic deviations $\boldsymbol{m}(\boldsymbol{r},t)$ around the equilibrium state $\boldsymbol{M}_0(\boldsymbol{r})$ and linearize the LLG equation with respect to $\boldsymbol{m}$. Assuming a harmonic time dependence $\boldsymbol{m}(\boldsymbol{r},t) = \boldsymbol{m}(\boldsymbol{r})\, e^{i\omega t}$, the weak-form LLG leads to a generalized eigenvalue problem for the complex eigenfrequency f, which is solved within the finite-element (FEM) framework of COMSOL. This approach naturally incorporates the full three-dimensional geometry and the inhomogeneous internal field landscape of the nanospike array.

Because the array is periodic in the plane of the membrane, Floquet–Bloch boundary conditions were applied in the two in-plane directions. According to Bloch's theorem, the dynamic magnetization satisfies:

$$\boldsymbol{m}_{\mathrm{dst}} = \boldsymbol{m}_{\mathrm{src}} \exp\big[i\boldsymbol{k}\cdot(\boldsymbol{r}_{\mathrm{dst}} - \boldsymbol{r}_{\mathrm{src}})\big], \tag{6}$$

where $\boldsymbol{m}_{\mathrm{src}}$ and $\boldsymbol{m}_{\mathrm{dst}}$ denote the dynamic magnetization at two corresponding points on opposite faces of the unit cell, $\boldsymbol{k} = (k_x, k_y)$ is the in-plane Bloch wavevector, and $\boldsymbol{r}_{\mathrm{dst}} - \boldsymbol{r}_{\mathrm{src}}$ is the in-plane lattice translation vector.

For translations along the primitive lattice vectors, Eq. (6) reduces to the phase-continuity conditions

$$\boldsymbol{M}(x + a,\, y,\, z) = \boldsymbol{M}(x,\, y,\, z)\, e^{ik_x a}, \tag{7}$$

$$\boldsymbol{M}(x,\, y + a,\, z) = \boldsymbol{M}(x,\, y,\, z)\, e^{ik_y a}, \tag{8}$$

where a is the in-plane lattice constant. Free boundary conditions were applied along the thickness direction z, allowing for the formation of surface-localized and apex-confined spin-wave modes at the topography of the nanospikes and ridges.

The resulting weak-form eigenvalue problem was solved for a discrete set of $\boldsymbol{k}$ values sampling the first Brillouin zone of the square lattice,

$$\boldsymbol{k} \in \left[0, \frac{\pi}{a}\right], \tag{9}$$

along the experimentally relevant directions, in particular [1 0] and [1 1]. This procedure provides the spin-wave dispersion relation $f(\boldsymbol{k})$ together with the corresponding spatial profiles of the dynamic magnetization.

VI. MAGNETIC FIELD DEPENDENCE IN BLS

These measurements were performed at the fixed wave vector $k_{\rm nom} = 4.1\,{\rm rad}/\mu{\rm m}$, corresponding to an incidence angle of 10°. This choice was made because certain peaks are not detected at $k_{\rm nom} = 0$ (normal light incidence), as observed in Figure 2c of the main text, whereas they become visible at $k_{\rm nom} = 4.1\,{\rm rad}/\mu{\rm m}$. Moreover, at normal light incidence, the high-intensity back reflected light beam enters the Fabry–Perot interferometer (FPI), which introduces significant noise into the spectra, despite the polarization analysis of light.

In this work, we perform measurements in magnetic fields ranging from −50 to +50 mT (Supporting Fig. 8). The low-field range is chosen to be able to track effects related to the magnetization following the curved profile of the template. When the field is applied along the [1 0] direction, a change in the slope is observed at approximately 17 mT, whereas this change is not visible for the case when the field is applied along the [1 1] direction. A systematic investigation of spin-wave properties at higher magnetic fields deserve a separate study.

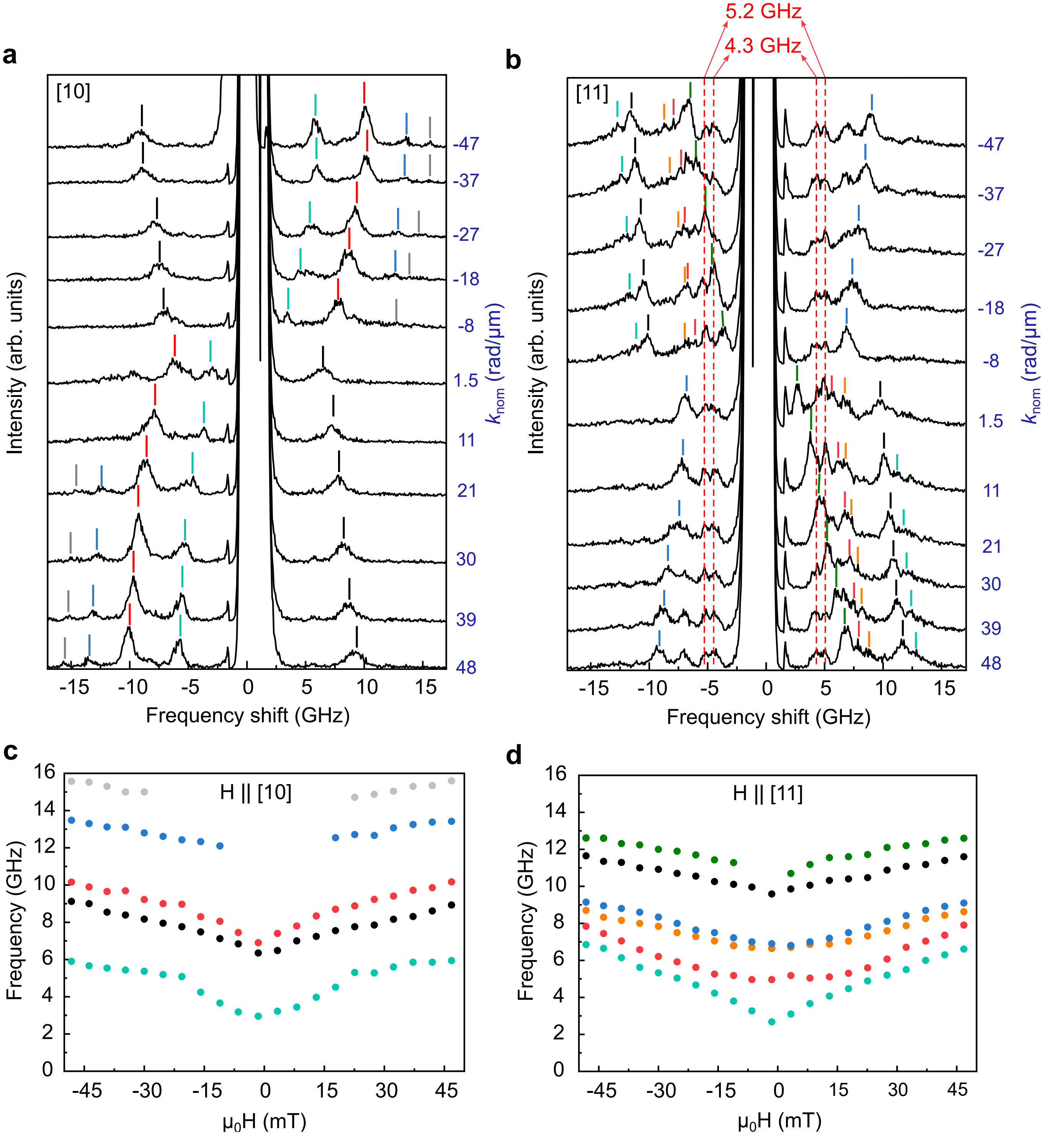

Supporting Fig. 8. Sequences of BLS spectra measured as $|k_{\mathrm{nom}}| = 4.1\,\mathrm{rad}/\mu\mathrm{m}$ as a function of the magnetic field H applied along (a) [1 0] and (b) [1 1] in-plane directions. The labels of each spectrum correspond to the magnetic field strength expressed in mT. The main peaks of the spectra are marked by vertical segments of different colors to serve as a visual guide for tracking the frequency evolution of the peaks in panels (c) and (d). In panel (b), the peaks at 4.30 GHz and 5.20 GHz, which remain constant as a function of magnetic field, are indicated by dotted red vertical lines and are attributed to be of a nonmagnetic origin. Panels (c) and (d) present the measured spin-wave frequency as a function of the applied magnetic field when it is parallel to [1 0] and [1 1] direction of the lattice.

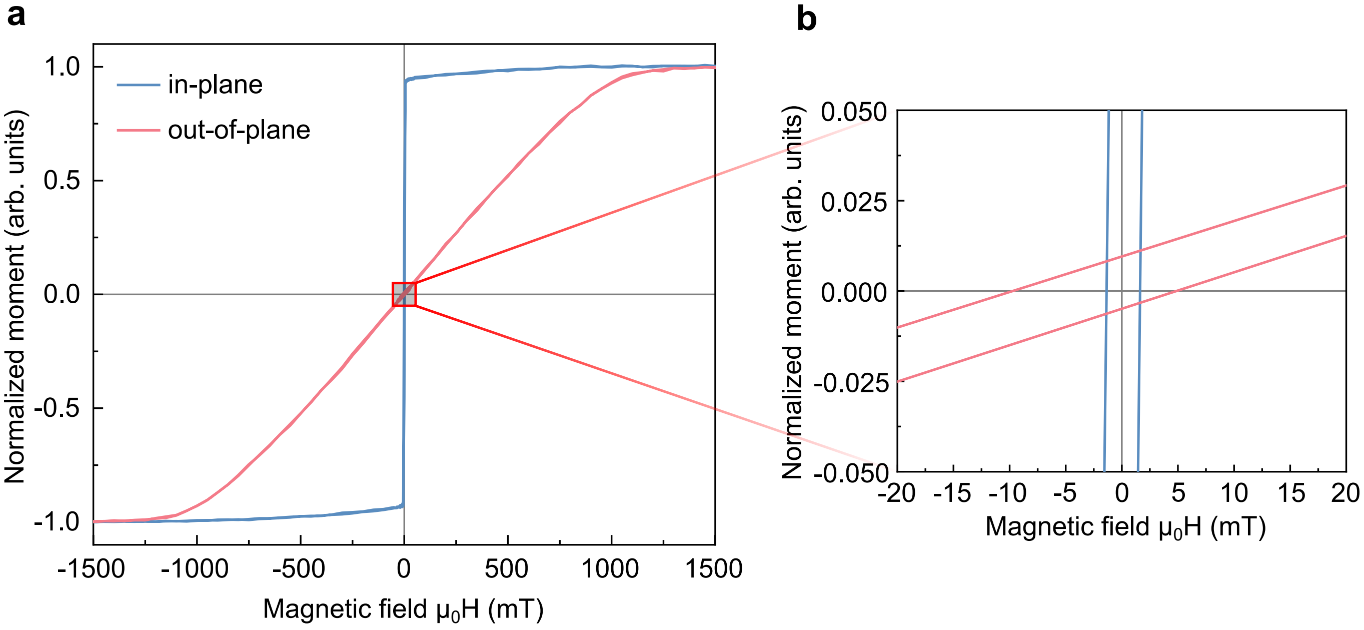

Supporting Fig. 9. (a) Magnetic hysteresis loop of the 30-nm-thick Permalloy reference film sputtered onto a SiO_2 planar substrate. (b) Zoomed-in view highlighting the loop opening at low magnetic fields. For the in-plane configuration (blue loop), the magnetic field was applied parallel to the film surface, whereas for the out-of-plane measurement (pink curve) it was oriented perpendicular to the surface. M(H) loops were obtained at room temperature using MicroSence EZ7 vector Vibrating Sample Magnetometer (VSM).

REFERENCES

[1] O. Bezsmertna, R. Xu, O. Pylypovskyi, D. Raftrey, A. Sorrentino, J. A. Fernandez-Roldan, I. Soldatov, D. Wolf, A. Lubk, R. Schäfer, P. Fischer, and D. Makarov, Magnetic solitons in hierarchical 3d magnetic nanoarchitectures of nanoflower shape, Nano Letters **24**, 15774 (2024).

[2] R. Xu, Z. Zeng, and Y. Lei, Well-defined nanostructuring with designable anodic aluminum oxide template, Nature Communications **13**, 10.1038/s41467-022-30137-6 (2022).

[3] A. Sorrentino, J. Nicolás, R. Valcárcel, F. J. Chichón, M. Rosanes, J. Avila, A. Tkachuk, J. Irwin, S. Ferrer, and E. Pereiro, Mistral: a transmission soft x-ray microscopy beamline for cryo nano-tomography of biological samples and magnetic domains imaging, Journal of Synchrotron Radiation **22**, 1112 (2015).

[4] A. E. Herguedas-Alonso, J. Gómez Sánchez, C. Fernández-González, A. Sorrentino, S. Ferrer, E. Pereiro, and A. Hierro-Rodriguez, Martapp: software for the processing and reconstruction of synchrotron-radiation-based magnetic tomographies, Journal of Synchrotron Radiation **32**, 1095 (2025).

[5] O. M. Volkov, D. Wolf, O. V. Pylypovskyi, A. Kákay, D. D. Sheka, B. Büchner, J. Fassbender, A. Lubk, and D. Makarov, Chirality coupling in topological magnetic textures with multiple magnetochiral parameters, Nature Communications **14**, 10.1038/s41467-023-37081-z (2023).

[6] J. Sandercock, Light scattering in solids iii, Topics in Applied Physics 10.1007/3-540-11513-7 (1982).

[7] R. Damon and J. Eshbach, Magnetostatic modes of a ferromagnet slab, Journal of Physics and Chemistry of Solids **19**, 308 (1961).

[8] A. Korniienko, V. Kravchuk, O. Pylypovskyi, D. Sheka, J. van den Brink, and Y. Gaididei, Curvature induced magnonic crystal in nanowires, SciPost Physics **7**, 10.21468/scipostphys.7.3.035 (2019).

[9] J. R. Sandercock and W. Wettling, Light scattering from surface and bulk thermal magnons in iron and nickel, Journal of Applied Physics **50**, 7784 (1979).

[10] G. Carlotti and G. Gubbiotti, Magnetic properties of layered nanostructures studied by means of brillouin light scattering and the surface magneto-optical kerr effect, Journal of Physics: Condensed Matter **14**, 8199 (2002).

[11] Y. Gaididei, V. P. Kravchuk, F. G. Mertens, O. V. Pylypovskyi, A. Saxena, D. D. Sheka, and O. M. Volkov, Localization of magnon modes in a curved magnetic nanowire, Low Temperature Physics **44**, 634 (2018).

[12] R. E. Camley, P. Grünberg, and C. M. Mayr, Stokes—anti-Stokes asymmetry in brillouin scattering from magnons in thin ferromagnetic films, Physical Review B **26**, 2609 (1982).

[13] S. Tacchi, F. Montoncello, M. Madami, G. Gubbiotti, G. Carlotti, L. Giovannini, R. Zivieri, F. Nizzoli, S. Jain, A. O. Adeyeye, and N. Singh, Band diagram of spin waves in a two-dimensional magnonic crystal, Physical Review Letters **107**, 127204 (2011).

[14] S. Tacchi, G. Duerr, J. W. Klos, M. Madami, S. Neusser, G. Gubbiotti, G. Carlotti, M. Krawczyk, and D. Grundler, Forbidden band gaps in the spin-wave spectrum of a two-dimensional bicomponent magnonic crystal, Physical Review Letters **109**, 137202 (2012).

[15] G. Gubbiotti, A. Sadovnikov, E. Beginin, S. Nikitov, D. Wan, A. Gupta, S. Kundu, G. Talmelli, R. Carpenter, I. Asselberghs, I. P. Radu, C. Adelmann, and F. Ciubotaru, Magnonic band structure in vertical meander-shaped $Co_{40}Fe_{40}B_{20}$ thin films, Physical Review Applied **15**, 014061 (2021).

[16] N. Challab, A. D. Aboumassound, F. Zighem, D. Faurie, and M. Haboussi, Micromagnetic modeling of nanostructures subject to heterogeneous strain fields, Journal of Physics D: Applied Physics **52**, 355004 (2019).

[17] M. Khelif, S. Chiroli, N. Challab, D. Faurie, M. Haboussi, and F. Zighem, Stress gradient effects on magnetic eigenmodes in ferromagnetic nanobars: a micromagnetic approach to magnetoelastic coupling, Journal of Physics D: Applied Physics **58**, 475001 (2025).

[18] N. Challab, F. Zighem, D. Faurie, M. Haboussi, M. Belmeguenai, P. Lupo, and A. O. Adeyeye, Local stiffness effect on ferromagnetic response of nanostructure arrays in stretchable systems, physica status solidi (RRL) – Rapid Research Letters **13**, 1800509 (2019), https://onlinelibrary.wiley.com/doi/pdf/10.1002/pssr.201800509.

[19] S. Chiroli, D. Faurie, M. Haboussi, A. O. Adeyeye, and F. Zighem, Magnetization dynamics of elastically strained nanostructures studied by coupled micromagnetic-mechanical simulations, Phys. Rev. B **108**, 024406 (2023).

[20] S. Chiroli, D. Faurie, A. O. Adeyeye, and F. Zighem, Tailoring band structures in 2d antidot magnonic crystals through heterogeneouselastic straining, under review.
